# Modified Colon Leakage Score to Predict Anastomotic Leakage in Patients Who Underwent Left-Sided Colorectal Surgery

**DOI:** 10.3390/jcm8091450

**Published:** 2019-09-12

**Authors:** Seung Up Yang, Eun Jung Park, Seung Hyuk Baik, Kang Young Lee, Jeonghyun Kang

**Affiliations:** 1 Department of Surgery, Gangnam Severance Hospital, Yonsei University College of Medicine, Seoul 06273, Korea; AURAS@yuhs.ac (S.U.Y.); camp79@yuhs.ac (E.J.P.); whitenoja@yuhs.ac (S.H.B.); 2 Department of Surgery, Severance Hospital, Yonsei University College of Medicine, Seoul 03722, Korea; kylee117@yuhs.ac

**Keywords:** anastomotic leakage, rectal neoplasms, colorectal cancer, area under the curve, LASSO

## Abstract

Colon leakage score (CLS) was introduced as a clinical tool to predict anastomotic leakage (AL) in patients who underwent left-sided colorectal surgery, but its clinical validity has not been widely studied. We evaluated the clinical utility of CLS and developed a modified CLS (m-CLS). In total, 566 patients who underwent left-sided colorectal surgery were enrolled and categorized into training (*n* = 396) and validation (*n* = 170) sets via random sampling. Using CLS variables, the least absolute shrinkage and selection operator (LASSO) regression model was applied for variable selection and predictive signature building in the training set. The model’s performance was validated in the validation set. The predictive powers of m-CLS and CLS were compared by the area under the receiver operating characteristic (AUROC) curve in the overall group. Twenty-three AL events (4.1%) were noted. The AL group had a significantly higher mean CLS than the No Leakage group (12.5 vs. 9.6, *p* = 0.001). Five clinical variables were selected and used to generate m-CLS. The predictive performance of m-CLS was similar in training and validation sets (AUROC 0.838 vs. 0.803, *p* = 0.724). In the overall set, m-CLS was significantly predictive of AL and performed better than CLS (AUROC 0.831 vs. 0.701, *p* = 0.008). In conclusion, LASSO-model-generated m-CLS could predict AL more accurately than CLS.

## 1. Introduction

Anastomotic leakage (AL) is one of the most serious and devastating post-operative complications of colorectal cancer surgery. AL has an incidence of 3–27% and a mortality rate of 25–37% [1,2,3,4,5,6]. It is an adverse risk factor for long-term outcomes in these patients [7,8,9,10]. Securing an adequate blood supply for the remaining bowel and a tension-free anastomosis are the most important principles of AL reduction. Splenic flexure mobilization and low ligation, which preserve the left colic artery for the remaining bowel, are procedures often performed for this purpose in a left-sided colorectal surgery, although there is still some debate on its role [11,12]. An air leak test and the use of an indwelling drainage tube are additional ways of potentially reducing AL [13,14,15,16,17,18].

In addition, the surgeon often has to decide whether to pursue either a protective ileostomy or a colostomy. It is believed that while diversion itself cannot diminish AL, it can reduce the severity of AL-induced complications [19,20]. However, it can cause morbidity, which cannot be ignored. The complications during diversion, especially in diverting ileostomy, include dehydration and stoma prolapse, which occur in 11–43% of cases [21,22,23,24,25]. Even at the time of reversing the stoma, the complication rate was reported as 14–33% and, in several cases, the permanent reversal of diversion is impossible [21,26,27,28,29,30]. Therefore, the decision to perform a diversion must be made prudently; however, to date, there have been no reliable indications available.

The colon leakage score (CLS) was initially suggested to predict the risk of AL in left-sided surgeries by Dekker and colleagues in 2011 [31]. The CLS was composed of several clinical parameters, based on previous studies, and was calculated as a numeric score ranging from 0 to 43. Although few studies have validated the efficacy of CLS in patients with colorectal cancer [32,33], the clinical significance of its use has undergone limited evaluation.

Recently, it was reported that the surgeon perception of treatment risk and benefit varied significantly, and a risk assessment or a risk calculator could lead surgeons to more accurate judgements of operative risks [34,35]. In the case of predicting AL for 83 patients with colon cancer, a simple anastomotic leak calculator was highly predictive of AL (area under the receiver-operating characteristic curve (AUROC), 0.84), as unlike the surgeon’s estimation (AUROC, 0.4) [36]. Accurate and clinically useful prediction models are gaining importance. Recently machine learning algorithms have been introduced that are actively applied in the clinical decision-making process [37,38]. We hypothesized that there might be some room to improve the predictive power of CLS by applying these algorithms.

Therefore, the aim of this study was to assess the clinical implications of the CLS in our patients and to improve the predictive power of this model.

## 2. Materials and Methods

### 2.1. Patients

The study group consisted of consecutive patients who underwent left-sided colorectal cancer surgeries at the Gangnam Severance Hospital, Yonsei University College of Medicine (Seoul, Republic of Korea) between August 2006 and March 2013. The patients were identified retrospectively from a prospectively-maintained database. The inclusion criterion was all patients with left-sided colorectal cancer (including left colon, sigmoid colon, rectosigmoid colon and rectum cancers) treated with a curative intent, irrespective of an elective or emergent surgery, in whom a primary anastomosis was performed. The exclusion criteria were patients who did not undergo primary anastomosis or R2 resection and patients with missing information, in which case the variables composing CLS could not be determined. This study was approved by Institutional Review Board of Gangnam Severance Hospital, Yonsei University College of Medicine (Seoul, Republic of Korea) (approval No. 3-2018-0326). Informed consent was waived for this retrospective study.

### 2.2. Surgery and Diagnosis of AL

Each of the enrolled patients underwent a different type of surgery based on the location of the tumor. A left hemicolectomy was done for tumors in the left or sigmoid colon, while an anterior resection or a low anterior resection was done for tumors in the distal or rectosigmoid colon. Patients with rectal cancer underwent a low anterior resection, coloanal anastomosis, or an intersphincteric resection. Patients who underwent a Hartmann operation or an abdominoperineal resection were excluded. The surgeries were performed using a consistent surgical principle. The standard surgical modality for patients with rectal cancer was either a total mesorectal excision or a tumor-specific mesorectal excision. For left-sided tumors, at the discretion of the surgeon, the inferior mesenteric artery was ligated at the level of its origin or just below the left colic artery. The decision to create a diverting ileostomy or colostomy was left to the discretion of the surgeon.

AL was initially suspected from clinical manifestations such as abdominal pain, fever, and discharge of pus or bowel contents through the abdominal drain causing peritonitis. It was confirmed by either laparotomy or imaging studies, such as computed tomography (CT) and X-ray.

### 2.3. Clinical Variables Used in Generating Modified CLS

CLS is a scoring system suggested by Dekker and colleagues [27]. The system combines the following 11 risk factors for AL: age, gender, the American Society of Anesthesiologists (ASA) grade, body mass index, history of intoxication, history of neoadjuvant therapy, history of emergency surgery, distance of anastomosis to the anal verge, requirement of additional procedures, amount of blood loss, and duration of the surgical procedure. Each variable is scored numerically, and the risk of AL is predicted on the basis of the total score, which ranges between 0 and 43 in the CLS model.

We collected data (used in calculating CLS) from our group, and only these variables were used in generating a new algorithm. In this study, the least absolute shrinkage and selection operator (LASSO) regression model was used to generate the predictive model [39]. In this model, the regression coefficients penalize the size of the parameters, which can remove unimportant variables. The LASSO regression model was applied for feature selection and predictive signature building, called the modified CLS (m-CLS). LASSO regression shrinks the coefficient estimates toward zero, with the degree of shrinkage dependent on an additional parameter, λ. To determine the optimal values of λ, a 10-time cross-validation was used, and we chose λ via the minimum criteria.

Our patients were divided into the following two groups via computer-generated random sampling; the training set and the validation set. The prediction model (m-CLS) was developed in the training set and validated in the validation set. The performance of the m-CLS, in comparison to the CLS, was measured by the AUROC analysis in the overall (training and validation) set.

### 2.4. Statistical Analysis

All statistical analyses were performed using IBM SPSS version 23.0 (IBM Corp., Armonk, NY, USA) and R version 3.5.1 (R-project, Institute for Statistics and Mathematics, Vienna, Austria). AUROC was used to determine the predictive value of CLS and m-CLS. The predictive value, estimated by AUROC, was classified as follows: >0.9: excellent, 0.8–0.9: good, 0.7–0.8: fair, 0.6–0.7: poor, 0.5–0.6: very poor. The optimal cut-off values were determined at the maxima of the Youden’s index and accuracy [40]. Categorical variables were analyzed using the Chi-square or the Fisher’s exact test, and continuous variables were analyzed using the Student’s *t* test. A *p* value < 0.05 was considered to indicate significance.

## 3. Results

### 3.1. Patient Characteristics According to the Anastomotic Leakage

**Figure 1:**
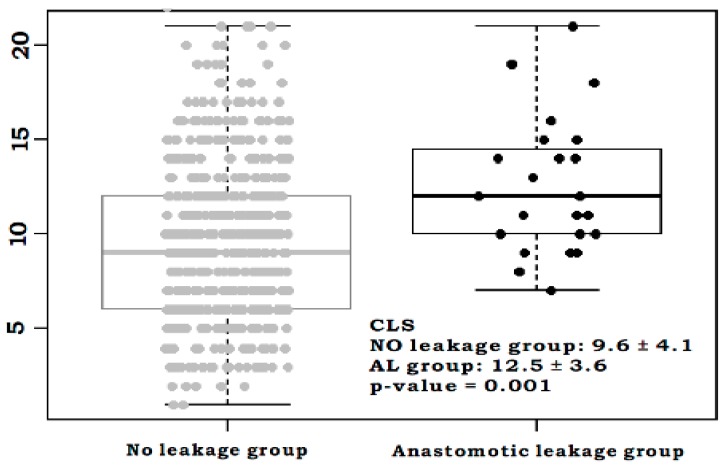
Comparison of colon leakage score according to the leakage status. The mean colon leakage score (CLS) was significantly higher in the anastomotic leakage group than the No leakage group (Mean ± standard deviation: 12.5 ± 3.6 in the AL group versus 9.6 ± 4.1 in the No leakage group, *p* = 0.001).

A total of 784 patients who underwent left-sided colorectal cancer surgeries were identified in the database, of which 566 were ultimately included in the analysis. Baseline patient characteristics according to the AL are shown in Table 1. While the overall AL rate was 4.1% (*n* = 23), the rate of low anastomosis level was significantly higher in the AL group. Alcohol intake and diversion rate were significantly higher in patients who had an AL versus those who did not. Mean CLS was significantly higher in the AL group than in the No Leakage group (12.5 vs. 9.6, *p* = 0.001) (Figure 1).

### 3.2. Comparison of Patient Characteristics between the Training Set and the Validation Set

By computer-generated random sampling, the patients were divided into the training set (396 patients) and the validation set (170 patients). There was no difference in the incidence of AL between the two sets (4.3% vs. 3.5%, *p* = 0.850). No difference in the clinicopathologic parameters were detected between the two groups (Table 2).

### 3.3. Feature Selection and Generation of Modified CLS

Of the clinical variables included in the CLS model, five potential predictors (based on the patients in the training set) were features with non-zero coefficients in the LASSO logistic regression model. These five parameters consisted of distance of anastomosis to anal verge, ASA grade 2, alcohol (3U/day), steroid (present use, excluding inhaler), and additional procedures. The combination of these parameters were presented as the modified CLS (Supplementary Figure S2). Distributions of the m-CLS in the overall set are given in the Supplementary File.

### 3.4. AUROC Comparison

**Figure 2:**
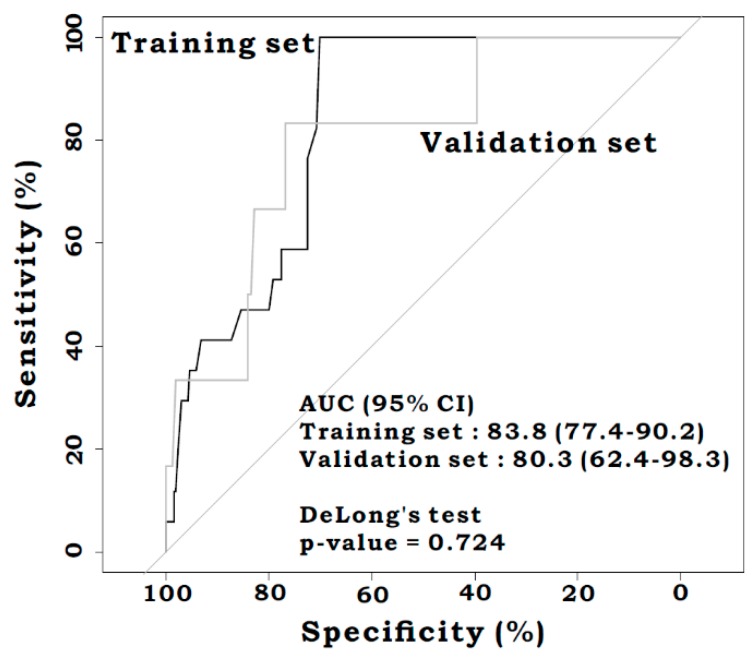
Receiver operating characteristic curves for the modified CLS (m-CLS) in the training set and the validation set. No significant difference of AUROC was seen between the train and validation sets (AUROC 0.838 versus 0.803, *p* = 0.724).

**Figure 3:**
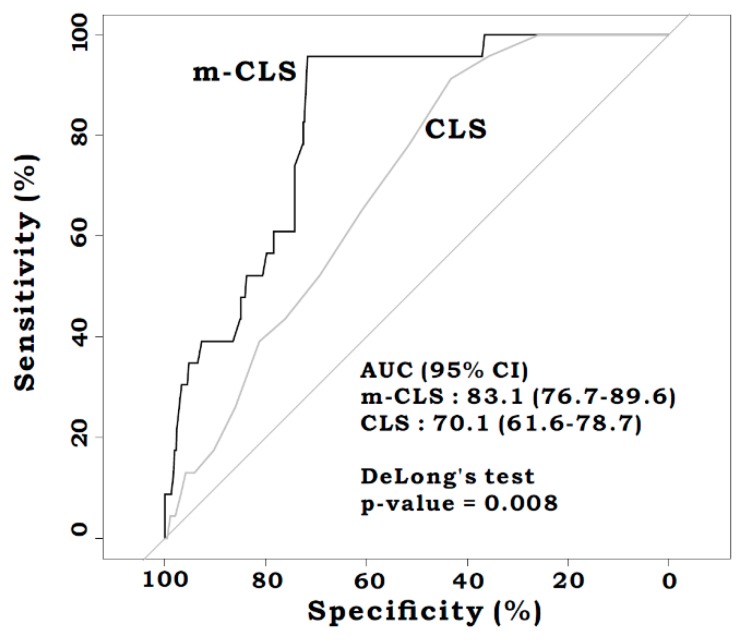
Receiver operating characteristic curves for the m-CLS and CLS in the overall (the train and the validation) set. This comparison revealed that the m-CLS performed better than CLS (AUROC 0.831 versus 0.701, *p* = 0.008).

There was no difference in the AUROC for m-CLS between the training and the validation sets [0.838 (95%CI: 0.774–0.902) vs. 0.803 (95%CI: 0.624–0.983), *p* = 0.724] (Figure 2). From the analysis of the overall set, the m-CLS was significantly predictive of AL, and better than the CLS [AUROC: 0.831 (95%CI: 0.767–0.896) vs. 0.701 (95%CI: 0.616–0.787), *p* = 0.008] (Figure 3). The sensitivity, specificity, positive predictive value (PPV), negative predictive value (NPV), and accuracy based on the specific cut-off values of the m-CLS and CLS are shown in Supplementary Tables S1 and S2. The statistically optimal cut-off value for the m-CLS was 0.055 (sensitivity: 0.957, specificity: 0.718, PPV: 0.126, NPV: 0.997, and accuracy: 0.728), and for the CLS was 8.5 (sensitivity: 0.913, specificity: 0.433, PPV: 0.064, NPV: 0.992, and accuracy: 0.452).

## 4. Discussion

This study demonstrated that m-CLS, which was developed using the LASSO logistic regression model derived from the clinical variables incorporated in the CLS, provides a more accurate prediction for the risk of AL than the CLS, in patients who underwent left-sided colorectal cancer surgeries.

The accuracy of CLS was initially reported to be quite high [AUROC, 0.95 (95% CI, 0.89–1.00)] [31]. Two studies performed subsequently to validate the CLS gave relatively good results, with the AUROC being 0.965 (95% CI, 0.913–1.00) and 0.80 (95% CI, 0.618–0.982) [32,33]. In this study, although the mean CLS was significantly higher in the AL group than in the No Leakage group, the predictive strength of CLS (AUROC 0.701) was not as high as was anticipated. Although it is difficult to reveal the exact reasons for this discrepancy, several factors may have been involved. An important CLS variable is the distance of anastomosis to the anal verge. This is a well-known clinical variable associated with the occurrence of AL in patients diagnosed with rectal cancer [41]. Nevertheless, it is sometimes difficult to accurately define the level of anastomosis, especially for left or sigmoid colon cancers in retrospective studies. In our study, since 27.8% of the patients were excluded, mainly due to a lack of this information, it may have led to a selection bias. Another factor that can be speculated upon is the low rate of patients with preoperative steroid use in our cohort. Although there is still some debate on the real impact of steroid use on AL risk and the definition of steroid use is variable, in previous prospective or retrospective studies, its rate in the pretreatment periods in colorectal surgeries ranged from 2.2 to 5.3% [42,43,44,45,46]. In this study, only four patients (0.7%) were classified as using steroids, although a lower rate of steroid use might be the specific characteristic of our patients. Considering the potential contribution of steroid use in AL occurrence, this relatively low rate seems to have worked in interrupting the accurate prediction of CLS.

Our study used the LASSO logistic regression model to determine whether the predictive power of m-CLS could be increased. The advantage of LASSO is that those variables from the patient data that are strongly associated with the prediction can be selected. Based on the comparison using ROC curves, we were able to confirm that the newly developed AL classifier (m-CLS) has a better predictive power than the CLS. Several AL risk prediction models have been developed for patients with colorectal cancer [33,47,48,49,50]. These predictive models would ultimately help surgeons decide whether to perform a diversion indirectly, by estimating a possibility of AL. However, neither our study nor previous studies could elucidate how these predictions would actually help reduce the AL rate in patients with colorectal cancer. Recently, a simple AL risk calculator was shown to have a better predictive power for AL than the surgeon’s estimation [36]. However, that study had several limitations, in that the number of included patients was relatively small and they all had colon cancer. Thus, we cannot confirm whether this calculator has any significant predictive role for patients with rectal cancer [36]. It is unclear whether using these predictive scoring systems rather than relying on the surgeon’s own experience to decide upon a diverting ileostomy would actually help in clinical practice. Further well-designed clinical trials are needed to confirm the validity of such predictive models.

There were several limitations in this study. This study is retrospective in nature, resulting in missing data that could result in a selection bias. In addition, this study was done in a single center using a relatively small sample size for prediction. The overall AL rate in our study was relatively low, thus it is questionable whether the predictive model we developed can be applied to other groups having higher AL rate as well. Our model was generated using the clinical outcomes of East Asian people. Since the patients’ characteristics differ with each hospital, as well as race, the risk prediction model cannot be applied globally. Although we tried to validate our model using our internal validation set, to overcome this limitation fundamentally, it requires external validation using an independent different population before it can be accepted for use in diverse situations. This study included patients who underwent left-sided colorectal cancer surgeries. AL is generally more frequent in patients with rectal cancer than in those with colon cancer. Therefore, the need for developing a predictive model for patients with rectal cancer is even greater. In our study, when we confined the subgroup to only patients with rectal cancer, the AUROC was significantly higher in the m-CLS model than in the CLS model [69.1 (95% CI, 58.2–79.9) vs. 54.4 (95% CI, 42.4–66.4), *p* = 0.037]. However, its overall accuracy in both predictive models was not satisfactory, demonstrating that a different predictive model for rectal cancer is required.

## 5. Conclusions

This study confirmed that m-CLS provides a more accurate prediction of AL than CLS in patients who have undergone left-sided colorectal cancer surgeries. Further research on the clinical efficacy of this prediction model in AL reduction is required. Besides, a prediction model specialized for patients with rectal cancer is warranted.

## Figures and Tables

**Table 1:** Comparison of patient characteristics between AL group and No leakage group.

		AL (*n* = 23) *n* (%)	No Leakage (*n* = 543) *n* (%)	*P*
Age (years)	Mean ± SD	60.8 ± 12.6	62.1 ± 10.9	0.574
Gender	Male	17 (73.9)	346 (63.7)	0.438
	Female	6 (26.1)	197 (36.3)	
ASA grade ^a^	I	16 (69.6)	272 (50.1)	0.076 ^b^
	II	4 (17.4)	226 (41.6)	
	III	3 (13)	44 (8.1)	
	IV	0	1 (0.2)	
BMI ^c^ (kg/m^2^)	Mean ± SD	23.7 ± 3.5	23.4 ± 3.2	0.672
Smoking	(any)	10 (43.5)	185 (34.1)	0.375
Alcohol	(3U/day)	9 (39.1)	109 (20.1)	0.036 ^b^
Steroids	(present use, excluding inhaler)	2 (8.7)	2 (0.4)	0.009 ^b^
Neoadjuvant therapy	Radiotherapy	0	3 (0.6)	>0.999 ^b^
	Chemoradiation	5 (21.7)	69 (12.7)	0.207 ^b^
Emergency surgery	Obstruction	4 (17.4)	100 (18.4)	>0.999 ^b^
	Bleeding	0	8 (1.5)	>0.999 ^b^
	Perforation	0	12 (2.2)	>0.999 ^b^
Distance of anastomosis to anal verge (cm)	<5	10 (43.5)	110 (20.3)	<0.001 ^b^
	5–10	12 (52.2)	186 (34.3)	
	>10	1 (4.3)	247 (45.5)	
Additional procedures		1 (4.3)	139 (25.6)	0.023
Blood loss (mL)	Mean ± SD	282.6 ± 280.6	287.9 ± 455.5	0.933
Duration of operation (min)	Mean ± SD	294.5 ± 76.8	272.6 ± 104.8	0.322
Diversion		2 (8.7)	4 (0.7)	0.021 ^b^
Tumor location	Colon	1 (4.3)	249 (45.9)	<0.001
	Rectum	22 (95.7)	294 (54.1)	
CLS ^d^	Mean ± SD	12.5 ± 3.6	9.6 ± 4.2	0.001
Surgery type	Open	7 (30.4)	175 (32.2)	0.458
	Laparoscopy	8 (34.8)	239 (44)	
	Robot	8 (34.8)	129 (23.8)	

AL: anastomotic leakage; SD: standard deviation; ^a^: American society of anesthesiology; ^b^: Fisher’s exact test; ^c^: Body mass index; ^d^: Colon leakage score.

**Table 2:** Patient demographics and operative outcomes between the training set and the validation set.

		Training Set (*n* = 396) *n* (%)	Validation Set (*n* = 170) *n* (%)	*P*
Age (years)	Mean ± SD	62.5 ± 10.8	61.2 ± 11.5	0.186
Gender	Male	251 (63.4)	112 (65.9)	0.637
	Female	145 (36.6)	58 (34.1)	
ASA grade ^a^	I	195 (49.2)	93 (54.7)	0.417
	II	169 (42.7)	61 (35.9)	
	III	31 (7.8)	16 (9.4)	
	IV	1 (0.3)	0	
BMI ^b^ (kg/m^2^)	Mean ± SD	23.6 ± 3.1	23.1 ± 3.3	0.102
Smoking	(any)	135 (34.1)	60 (35.3)	0.857
Alcohol	(3U/day)	75 (18.9)	43 (25.3)	0.111
Steroids	(present use, excluding inhaler)	3 (0.8)	1 (0.6)	>0.999
Neoadjuvant therapy	Radiotherapy	1 (0.3)	2 (1.2)	0.449
	Chemoradiation	51 (12.9)	23 (13.5)	0.941
Emergency surgery	Obstruction	76 (19.2)	28 (16.5)	0.517
	Bleeding	7 (1.8)	1 (0.6)	0.446 ^b^
	Perforation	10 (2.5)	2 (1.2)	0.482
Distance of anastomosis to anal verge (cm)	<5	85 (21.5)	35 (20.6)	0.575
	5–10	143 (36.1)	55 (32.4)	
	>10	168 (42.4)	80 (47.1)	
Additional procedures		96 (24.2)	44 (25.9)	0.758
Blood loss (mL)	Mean ± SD	280.3 ± 421.4	304.9 ± 510.2	0.580
Duration of operation (min)	Mean ± SD	275.9 ± 109.5	267.6 ± 89.1	0.345
Diversion		3 (0.8)	3 (1.8)	0.532
Tumor location	Colon	206 (52)	89 (52.4)	>0.999
	Rectum	190 (48)	81 (47.6)	
CLS ^c^	Mean ± SD	9.8 ± 4	9.7 ± 4.6	0.942
Anastomotic leakage		17 (4.3)	6 (3.5)	0.850
Surgery type	Open	131 (33.1)	51 (30)	0.656
	Laparoscopy	168 (42.4)	79 (46.5)	
	Robot	97 (24.5)	40 (23.5)	

SD: Standard Deviation; ^a^: American society of anesthesiology; ^b^: Body mass index; c: Colon leakage score.

## Supplementary Materials

The following are available online at https://www.mdpi.com/2077-0383/8/9/1450/s1, Figure S1: Selection of significant parameters in clinicopathologic variables based on the CLS in the training set and definition of linear predictor, Figure S2: Distribution of colon leakage score and modified CLS in overall patients, Figure S3: Receiver operating characteristic curves for the m-CLS and the CLS in patients with rectal cancer (*n* = 271) Subset analysis of rectal cancer patients revealed that the m-CLS performed better than CLS (AUROC 0.691 in m-CLS versus 0.544 in CLS, *p* = 0.037), Table S1: Sensitivity, specificity, PPV, NPV, and accuracy of predicted probability using the CLS at each cut-off point from 5 to 15, in units of 1, Table S2: Sensitivity, specificity, PPV, NPV, and accuracy of predicted probability using the m-CLS at each cut-off point from 3% to 15%, in units of 1%.
