# Supplementary material for: Modified Colon Leakage Score to Predict Anastomotic Leakage in Patients Who Underwent Left-Sided Colorectal Surgery
Source: J Clin Med. 2019 Sep 12;8(9):1450. doi: 10.3390/jcm8091450 (PMC6780090; doi:10.3390/jcm8091450)
Supplement: Supplementary file 1 [file jcm-08-01450-s001.zip › jcm-580671-SI.docx]

Figure S1. Selection of significant parameters in clinicopathologic variables based on the CLS in the training set and definition of linear predictor

| (A) Ten time cross validation for tuning parameter selection in the LASSO model | (B) LASSO coefficient profiles |
| --- | --- |
| 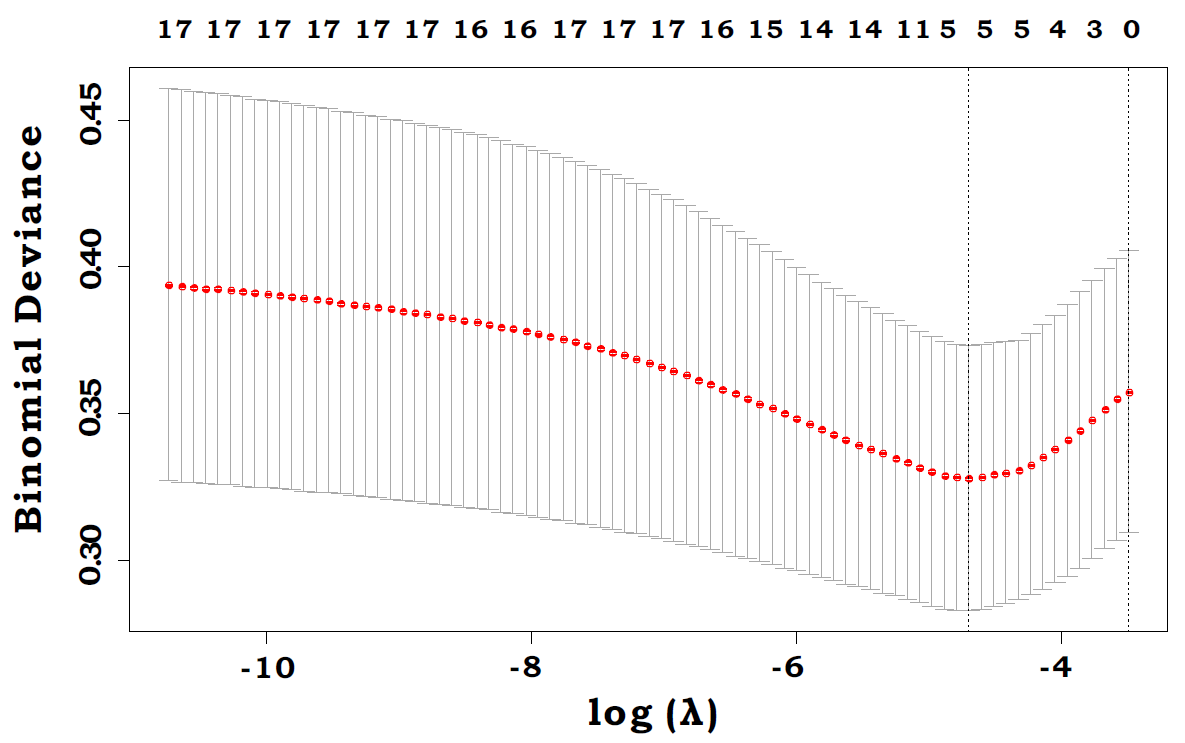 | 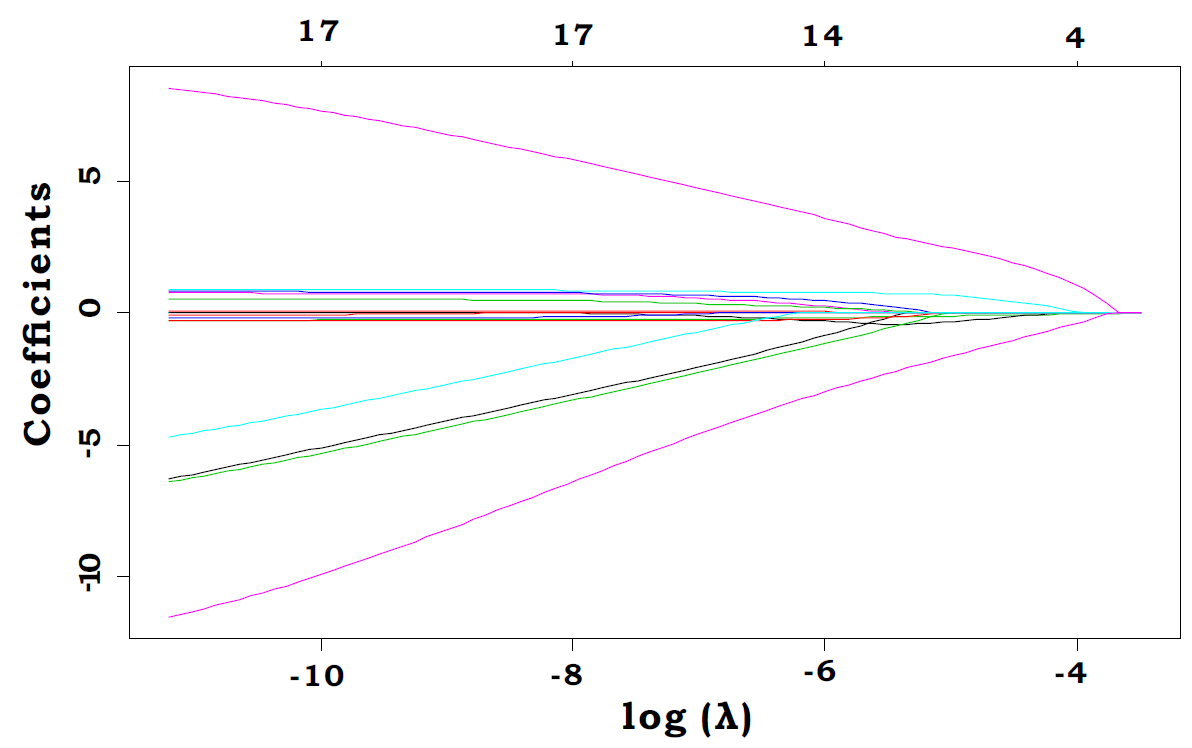 |

The least absolute shrinkage and selection operator method (LASSO) was used for regression of high dimensional predictors. The method uses an L1 penalty to shrink some regression coefficients to exactly zero.

(A) The binomial deviance curve was plotted versus log (λ), where λ is the tuning parameter. Solid vertical lines represent binomial deviance ± standard error (SE). The dotted vertical lines are drawn at the optimal values by using the minimum criteria and 1-SE criteria. Tuning parameter (λ) selection in the LASSO model used 10-fold cross-validation via minimum criteria. A value λ = 0.009137028 with log (λ) = -4.69542 was chosen.

(B) LASSO coefficient profiles of clinicopathologic variables. A coefficient profile plot was produced against the log (λ) sequence. The optimal tuning parameter resulted in five non-zero coefficients. Five parameters, Distance of anastomosis to anal verge, ASA grade2, alcohol (3U/day), steroid (present use, excluding inhaler), and additional procedures, with coefficients -0.09687268, -0.23044053, 0.53837188, 2.13571240, and -1.26541224 respectively, were selected in the LASSO model. The linear predictor was defined as Distance of anastomosis to anal verge x (-0.09687268) + ASA grade2 x (-0.23044053) + [alcohol (3U/day)] x 0.53837188 + [steroid (present use, excluding inhaler)] x 2.13571240 + additional procedures x (-1.26541224) – 2.12780965

Figure S2. Distribution of colon leakage score and modified CLS in overall patients

1. Colon leakage score


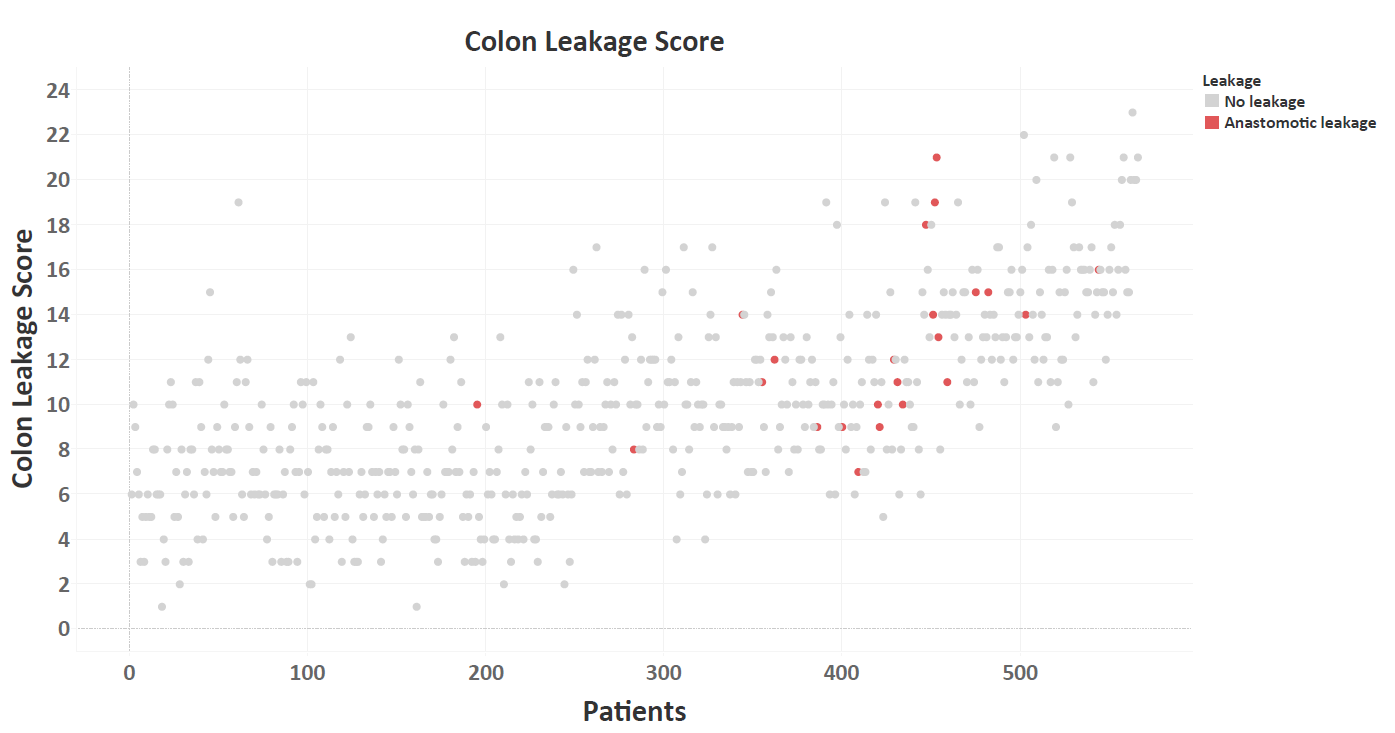


1. Modified Colon leakage score


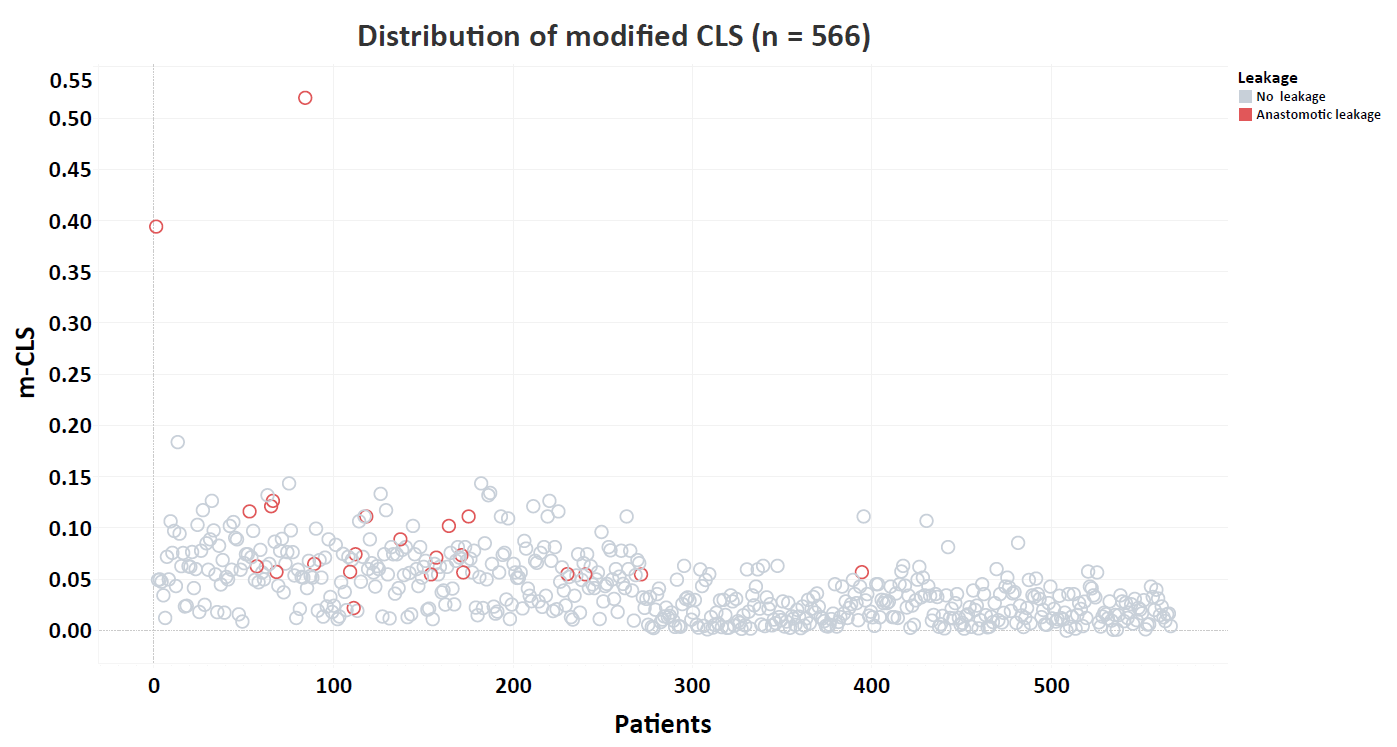


Figure S3. Receiver operating characteristic curves for the m-CLS and the CLS in patients with rectal cancer (n = 271) Subset analysis of rectal cancer patients revealed that the m-CLS performed better than CLS (AUROC 0.691 in m-CLS versus 0.544 in CLS, p=0.037)


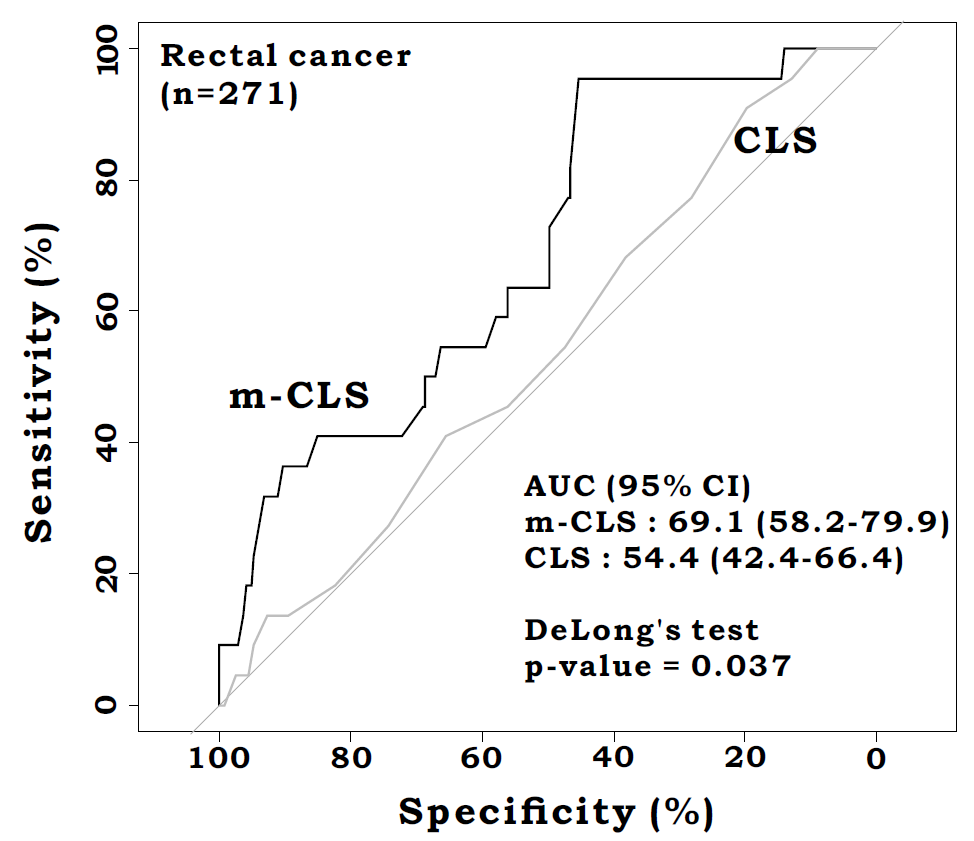


Table S1. Sensitivity, specificity, PPV, NPV, and accuracy of predicted probability using the CLS at each cut-off point from 5 to 15, in units of 1

| CLS | Sensitivity | Specificity | PPV | NPV | Accuracy | AUROC |
| --- | --- | --- | --- | --- | --- | --- |
| 5 | 1 | 0.096 | 0.045 | 1 | 0.133 | 0.548 |
| 6 | 1 | 0.155 | 0.048 | 1 | 0.189 | 0.577 |
| 7 | 1 | 0.258 | 0.054 | 1 | 0.288 | 0.629 |
| 8 | 0.957 | 0.357 | 0.059 | 0.995 | 0.382 | 0.657 |
| 9 | 0.913 | 0.433 | 0.064 | 0.992 | 0.452 | 0.673 |
| 10 | 0.783 | 0.516 | 0.064 | 0.982 | 0.527 | 0.649 |
| 11 | 0.652 | 0.610 | 0.066 | 0.976 | 0.611 | 0.631 |
| 12 | 0.522 | 0.694 | 0.067 | 0.972 | 0.687 | 0.608 |
| 13 | 0.435 | 0.762 | 0.072 | 0.970 | 0.749 | 0.599 |
| 14 | 0.391 | 0.812 | 0.081 | 0.969 | 0.795 | 0.602 |
| 15 | 0.261 | 0.860 | 0.073 | 0.965 | 0.836 | 0.560 |

PPV: positive predicted value

NPV: negative predicted value

Table S2. Sensitivity, specificity, PPV, NPV, and accuracy of predicted probability using the m-CLS at each cut-off point from 3% to 15%, in units of 1%

| Predicted probability (%) | Sensitivity | Specificity | PPV | NPV | Accuracy | AUROC |
| --- | --- | --- | --- | --- | --- | --- |
| 3 | 0.957 | 0.470 | 0.071 | 0.996 | 0.489 | 0.713 |
| 4 | 0.957 | 0.578 | 0.088 | 0.997 | 0.594 | 0.767 |
| 5 | 0.957 | 0.67 | 0.109 | 0.997 | 0.682 | 0.813 |
| 6 | 0.609 | 0.759 | 0.097 | 0.979 | 0.753 | 0.684 |
| 7 | 0.522 | 0.836 | 0.119 | 0.976 | 0.823 | 0.679 |
| 8 | 0.391 | 0.897 | 0.138 | 0.972 | 0.876 | 0.644 |
| 9 | 0.348 | 0.937 | 0.19 | 0.971 | 0.913 | 0.643 |
| 10 | 0.348 | 0.952 | 0.235 | 0.972 | 0.928 | 0.650 |
| 11 | 0.304 | 0.967 | 0.280 | 0.97 | 0.940 | 0.636 |
| 12 | 0.174 | 0.982 | 0.286 | 0.966 | 0.949 | 0.578 |
| 13 | 0.087 | 0.987 | 0.222 | 0.962 | 0.951 | 0.537 |
| 14 | 0.087 | 0.994 | 0.4 | 0.963 | 0.958 | 0.541 |
| 15 | 0.087 | 0.998 | 0.667 | 0.963 | 0.961 | 0.543 |

PPV: positive predicted value

NPV: negative predicted value
